# Understanding the variability of the proximal femoral canal: A computational modeling study

**DOI:** 10.1002/jor.25971

**Published:** 2024-09-18

**Authors:** Angelika Ramesh, Johann Henckel, Alister Hart, Anna Di Laura

**Affiliations:** ^1^ Department of Mechanical Engineering University College London Gower Street London UK; ^2^ Royal National Orthopaedic Hospital NHS Trust Brockley Hill Stanmore UK; ^3^ Institute of Orthopaedics and Musculoskeletal Science University College London Gower Street London UK; ^4^ Cleveland Clinic London London UK

**Keywords:** intramedullary femoral canal, principal component analysis, proximal femur, statistical shape modeling

## Abstract

Statistical shape modeling (SSM) offers the potential to describe the morphological differences in similar shapes using a compact number of variables. Its application in orthopedics is rapidly growing. In this study, an SSM of the intramedullary canal of the proximal femur was built, with the aim to better understanding the complexity of its shape which may, in turn, enhance the preoperative planning of total hip arthroplasty (THA). This includes the prediction of the prosthetic femoral version (PFV) which is known to be highly variable amongst patients who have undergone THA. The model was built on three dimensional (3D) models of 64 femoral canals which were generated from pelvic computed tomography images including the proximal femur in the field of view. Principal component analysis (PCA) was performed on the mean shape derived from the model and each segmented canal. Five prominent modes of variations representing approximately 84% of the total 3D variations in the population of shapes were found to capture variability in size, proximal torsion, intramedullary femoral anteversion, varus/valgus orientation, and distal femoral shaft twist/torsion, respectively. It was established that the intramedullary femoral canal is highly variable in its size, shape, and orientation between different subjects. PCA‐driven SSM is beneficial for identifying patterns and extracting valuable features of the femoral canal.

## 1 INTRODUCTION

Considering the growing use of uncemented stems in total hip arthroplasty (THA), the positioning of the prosthetic femoral component has become a vital point of discussion. Malpositioning of the stem can lead to prosthetic impingement, accelerated wear, and limited range of motion.^1^, ^2^ Unlike cemented fixation, which gives the surgeon more intraoperative control over the prosthetic femoral version (PFV),^3^, ^4^ uncemented THA allows limited intraoperative control due to the natural twist and bow of the intramedullary canal.^5^, ^6^ The main advantage of performing an uncemented THA is the reduction in operating room time since there is no need to prepare the bone cement.

The femoral version is conventionally measured using the external geometry of the femur and the knee joint. The relationship between the native femoral version (NFV) and the PFV has been reported to differ widely ranging from −20.7° to 21.5°, for an uncemented, straight tapered stem.^7^ Therefore, in uncemented THA, the NFV cannot be considered a reliable reference;^4^, ^8^ a better understanding of the geometry of the internal femoral canal will improve the planning of the PFV. This feature is currently only considered when designing custom stems.

Despite the preoperative and intraoperative attempts to accurately predict the version of femoral stems,^9^ the internal anatomy of the femur is less well understood. The intramedullary canal has not been extensively studied, and it does not have the same protruding landmarks and curvature as the external geometry of the femur.^10^, ^11^ Thus, in cases of uncemented THA, it is crucial to recognize patterns between the shape of the femoral canal and the achieved PFV, to predict the version of the femoral component.

The aim of the current study was to better understand the shape variations that characterize the intramedullary femoral canal which dictates the fit of the prosthetic stem. Our objective was to use statistical shape modeling (SSM) to identify the main modes of variation of the proximal femoral canal between different subjects. The null hypothesis was that there was no intersubject variability in the shape of the intramedullary femoral canal.

## 2 MATERIALS AND METHODS

### 2.1 Data collection—Training set

A total of 64 preoperative pelvic computed tomography (CT) scans of patients who subsequently underwent hip replacement with uncemented stems were used in this retrospective Cohort study (age range: 41–86 years, mean: 64 years; 31 males, 33 females). The mean height, weight, and BMI of this patient cohort was 170 cm (range: 152–188 cm), 82 kg (range: 50–113 kg), and 28 kg/m^2^ (range: 20–41 kg/m^2^), respectively.

The DICOM CT images were imported into Simpleware SCANIP Medical (Version 2022.3; Synopsys Inc.). Three dimensional (3D) models of the patient's intramedullary femoral canal were generated from each CT scan, using a semi‐automatic segmentation method. This involved a Boolean subtraction of a segmented model of the proximal femoral bone (with a cavity in regions representing the femoral canal), from a “completely filled” proximal femur, resulting in a segmentation of solely the canal. The default Hounsfield Unit (HU) values for a bone range from 230 to 3020 HU.^12^, ^13^ This was used for the threshold‐based segmentation for all cases. The same individual performed all segmentations for consistency in the workflow.

Each training case was prepared independently and made consistent for length (15 cm) by cropping the distal end of the canals longer than 15 cm to the desired length. It is worth noting that the preoperative CT scans that captured less than 15 cm of the proximal femur in the field‐of‐view were excluded from this study; this was done considering that the standard length of femoral stems used in primary THA is around 15 cm. The femoral canal shapes were filtered to smoothen the morphology and minimize surface roughness (by suppressing noise), using a Gaussian recursive filter. Any protruding features which were not found to be consistent among the full data set were removed in this way while retaining the main body/geometry of the intramedullary canal. The deviation between the unsmoothed and smoothed femoral canal models was quantified for a subset of ten surfaces using two metrics: Root‐Mean‐Square‐Error (RMSE) and Intersection over Union (IoU). These measure the geometric differences between the two surfaces; an RMSE below 1 and an IoU above 0.5 was considered satisfactory.^14^ The mean RMSE and IoU values measured were 0.42 and 0.91, respectively. This is suggestive of satisfactory smoothing without loss of crucial data.

Since each patient was positioned differently within the CT scanner, it was crucial to standardize the reference system of the femurs before they were aligned. A fixed coordinate system was defined for each canal, based on anatomical landmarks taken on the external geometry, namely the posterior condyles and the intertrochanteric crest, allowing for the training data set to be aligned in a well‐defined reference frame. This coordinate system is commonly used by orthopedic companies to define the femoral anteversion plane which is used for surgical planning.^15^

### 2.2 Point distribution model

**Figure 1:**
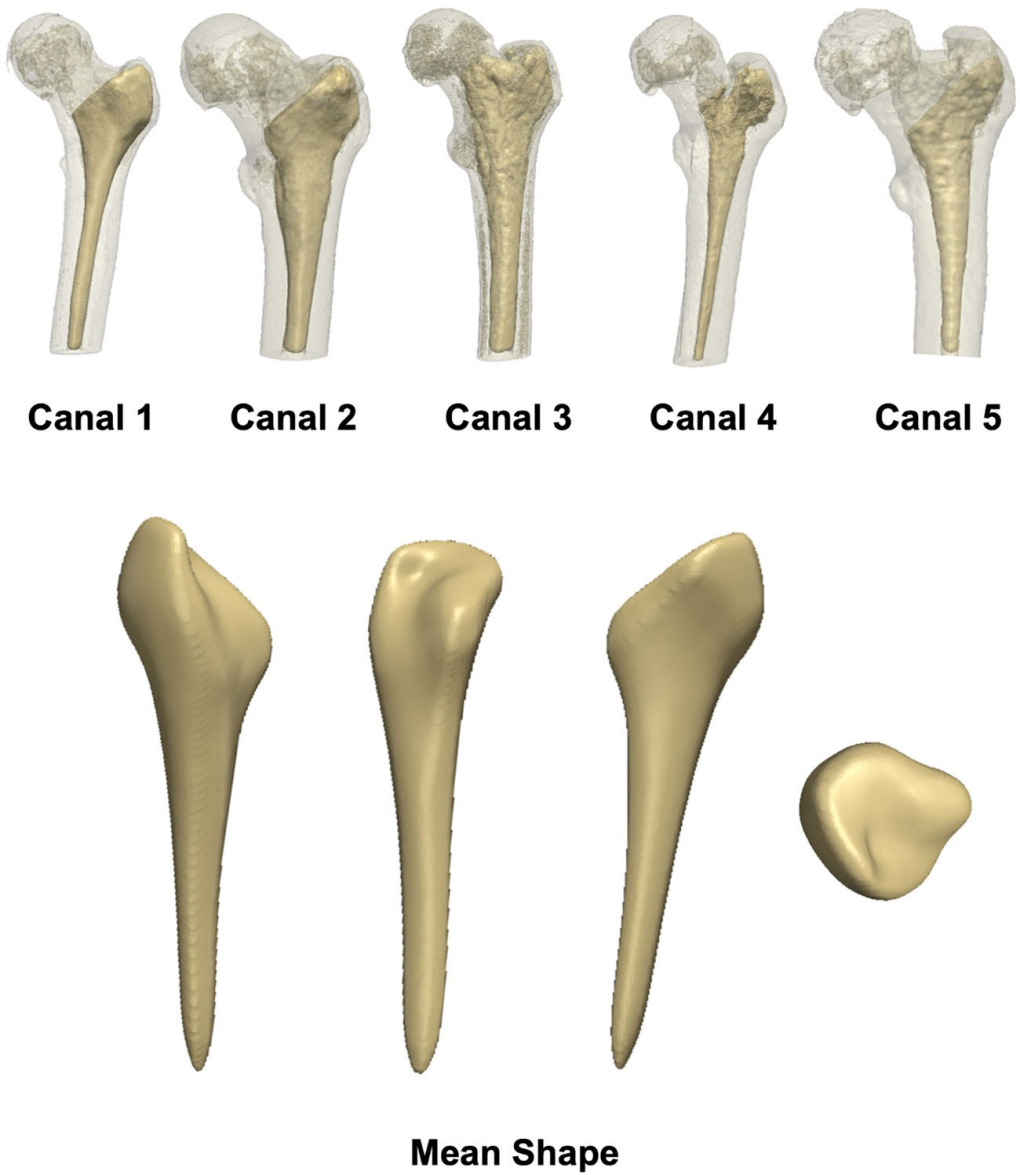
(Top row) The segmentations of the femoral canal for five of the cases used in this study, within the external geometry of the proximal femur, reveal noticeable variability. (Bottom row) Coronal, sagittal, and axial views of the mean shape which was computed mathematically from the total 64 canals on which the statistical shape modeling was built.

**Figure 2:**
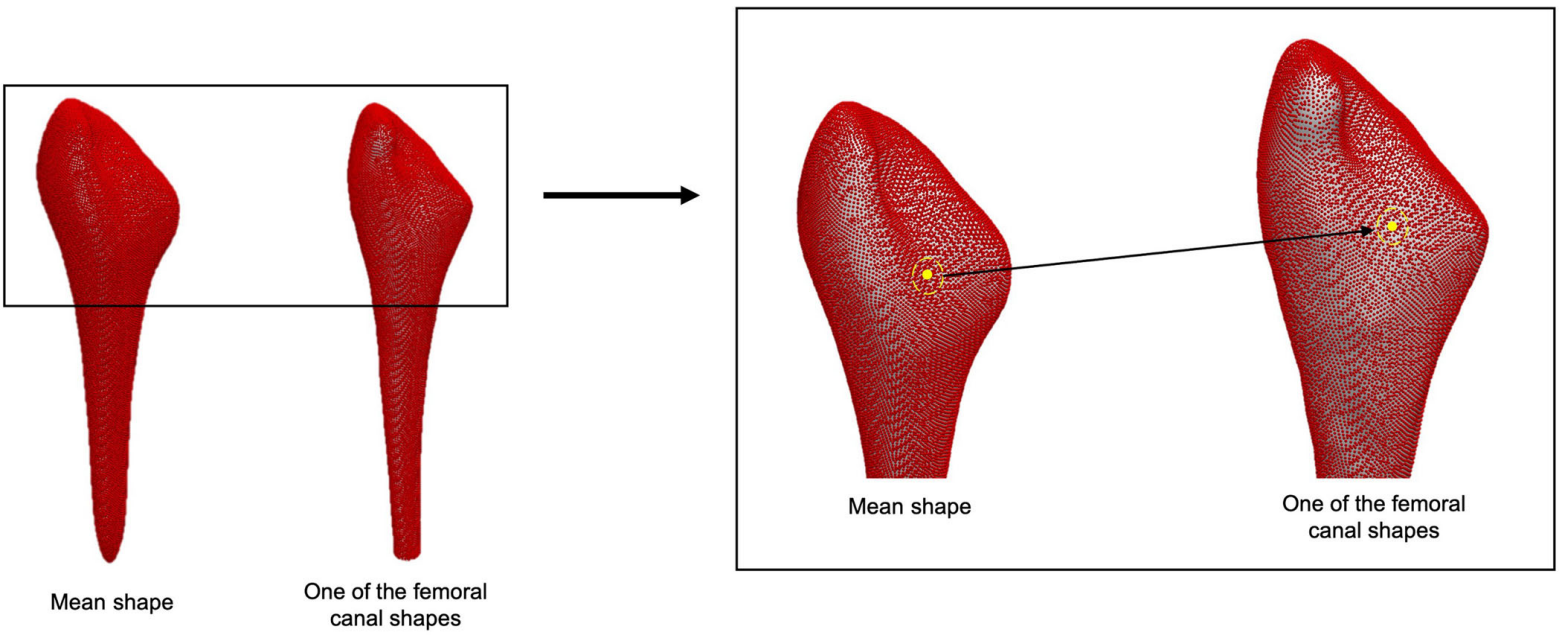
A figure illustrating the point distribution models generated from mapping each femoral canal onto the mean shape, using a set of equal numbers of points (point mapping). The mapping of a single point (in yellow) is highlighted.

A mean shape was computed; this is an average shape representation of the population (Figure 1). To investigate shape variations, a point mapping functionality was used which applied a rigid registration between each input surface and the target mean surface. This allowed for point correspondence to be determined. Through the point‐set representation of each femoral canal shape, the SSM is able to capture the relative positions of these points and identify patterns of shape variability among patients, as each shape was represented by an equal number of nodes (Figure 2).

### 2.3 Principal component analysis (PCA)

The point distribution models (PDM) generated for all 64 input canals were then used hereafter for the analysis. We used a PCA‐based dimensionality reduction technique to obtain a compact description of the shape variations between the models.

PCA was performed on the training set data. This tool allows for the directions of the highest shape variation to be extracted, also known as the modes or principal components (PCs). These eigenvectors are computed from the covariance matrix which displays the covariance values associated with all possible pairs of initial variables within the data set. The eigenvalues, representing the extent of projected change/variance in the corresponding direction, are also obtained from this matrix.

Once PCA was run, the primary modes of variation could be visualized by applying different weights to each mode, giving a unique shape for each combination of weights. A scree plot and a plot of the cumulative proportion of explained variance were generated for further analysis. This indicated how many modes are required to capture the majority (≥80%) of the variation in the input data set, since the principle component (PC) modes are ranked in order of contribution to the overall variance.

Perturbations of ±3 standard deviations (SD) from the mean were applied to each mode of variation, to visualize changes in size, shape, and alignment. The authors visually inspected the mean shape as the weight applied to each principal mode was varied, to assign that PC to an anatomical characteristic, by tracking how key features within the data set change between and within the modes.

**Figure 3:**
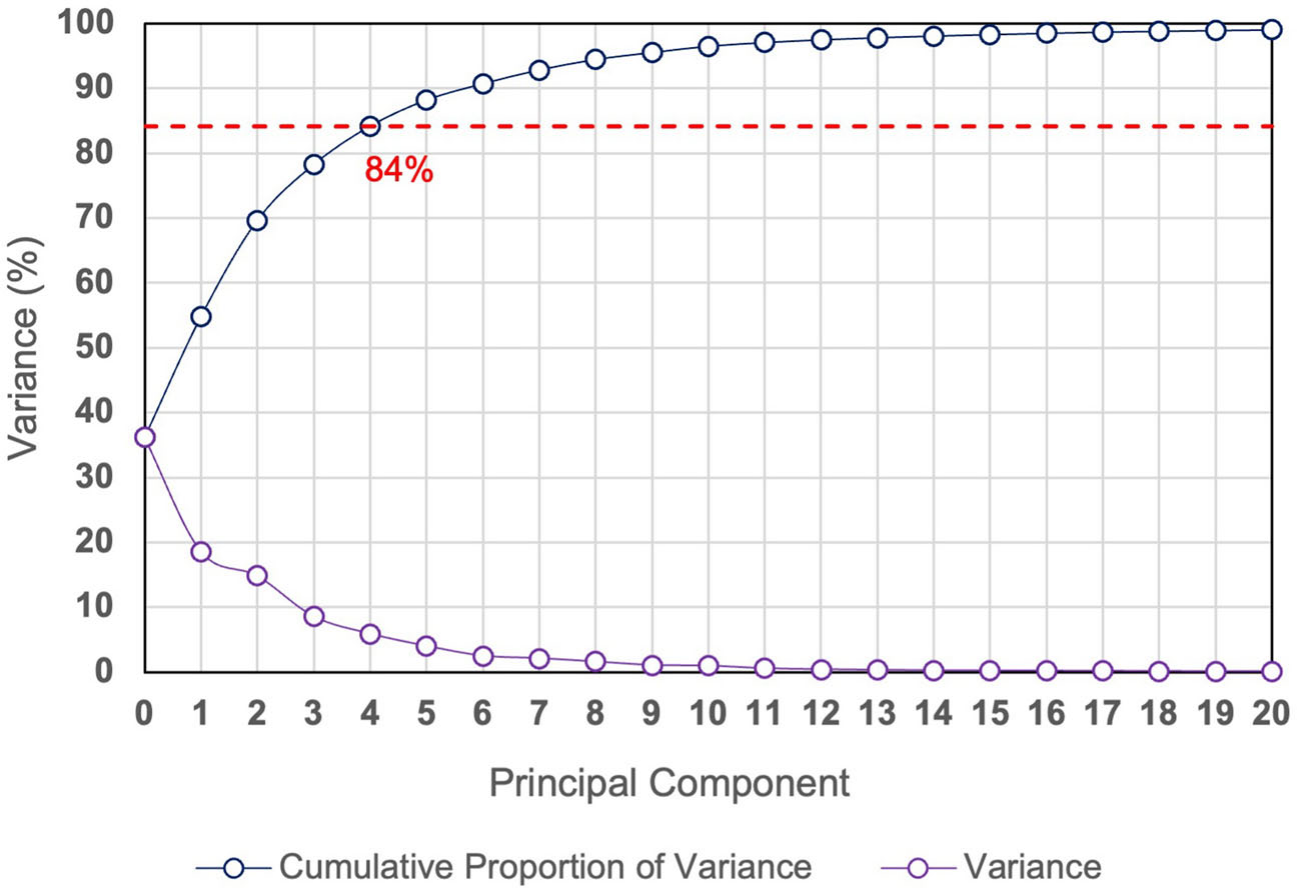
A scree plot showing the results of the principle component analysis; (1) the variance captured by each individual principal component, and (2) the cumulative proportion of explained variance.

To assess how many PCs are sufficient to capture a majority of the variability in the input data set used in this study, the scree plot was used (Figure 3). This plot shows that the first five principal components accounted for 84% of the total cumulative variance in the training data. This was assumed to be sufficient to cover most of the relevant features of the femoral canal shape, since the individual variance of mode 6 and onwards were below 5%. Hence, these were not included in the analysis. The modes are ranked in an order of decreasing contribution to the variation within the population data set, therefore the first five modes of variation are presented in detail in this paper hereafter.

The first five PCs were manipulated by ±3 SD and each manipulated shape was compared to the mean shape. To understand which anatomical characteristic was responsible for the majority of the variation in each mode (how the PC influences the overall shape of the canal), the femoral canal shapes were compared to the mean in the three anatomical views (axial, coronal, and sagittal).

## 3 RESULTS

### 3.1 Model evaluation

**Figure 4:**
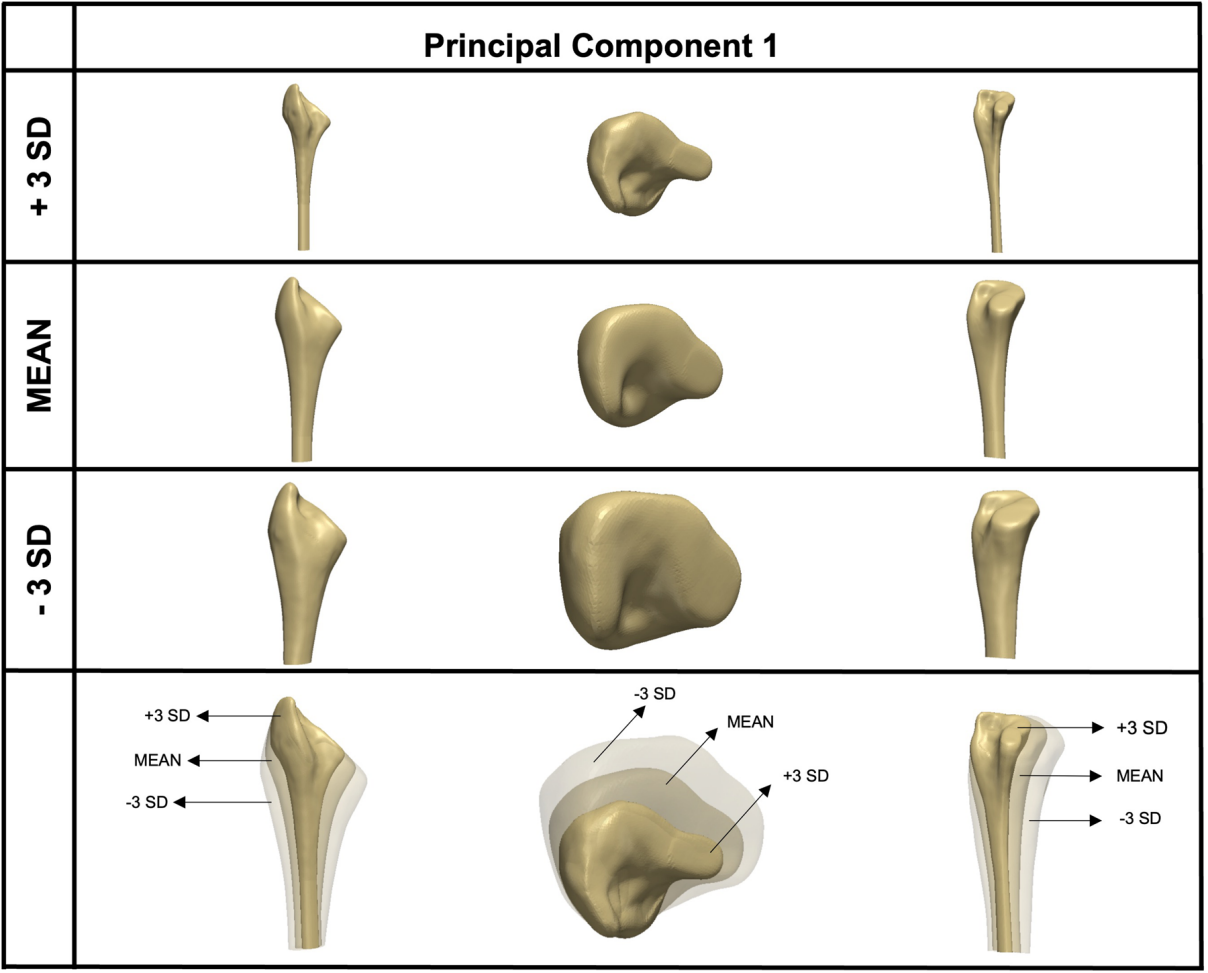
Analysis of principle component 1. The mean model is shown in the second row, along with ±3 standard deviation (SD) models (top and third row), revealing a change in size predominantly taking place. The left column shows an anteroposterior view, the middle column shows the axial view and the right column shows the lateral view of the femoral canal.

**Figure 5:**
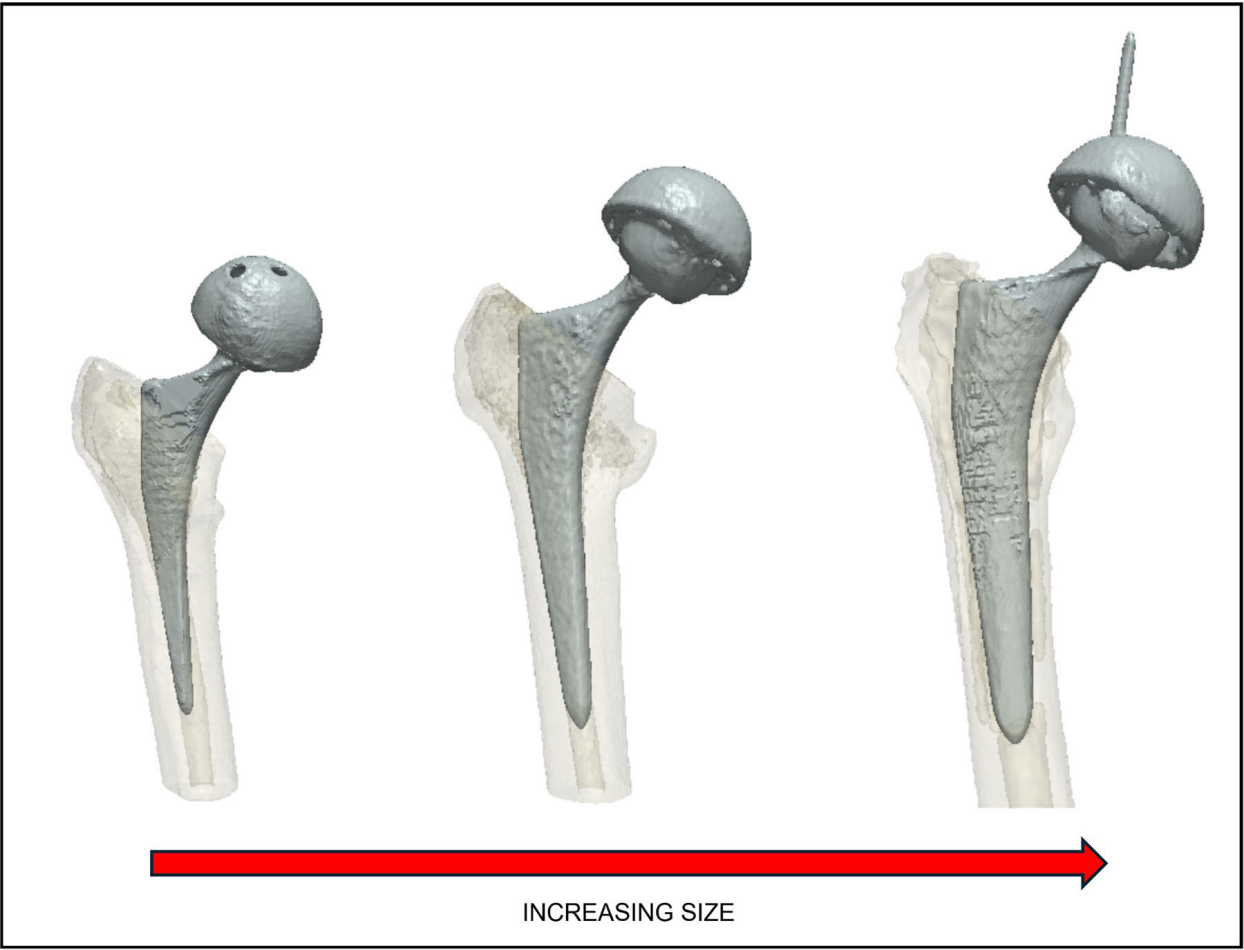
Different sized prosthetic femoral stems implanted in patients with different intramedullary canal geometry (size).

The first PC identifies the direction of greatest variance. Upon visual examination, size appeared to be the most prominently changing feature that this PC captures. But to accurately interpret this mode and to make a less subjective decision, the evolution of the mode was studied by taking feature‐based measurements. In other words, the mean shape was deformed by setting the weight of each PC from −3 SD to +3 SD, in equal intervals of +1, and taking volume measurements of the shape at each increment. The results were then plotted to identify in which mode size/volume is changing most significantly (steepest line of best fit). From this analysis, it was confirmed that the first PC represents variability in the size of the femoral canal (Figures 4 and 5) and accounted for 36% of the anatomical variation (eigenvalue = 3.8 × 10^5^).

**Figure 6:**
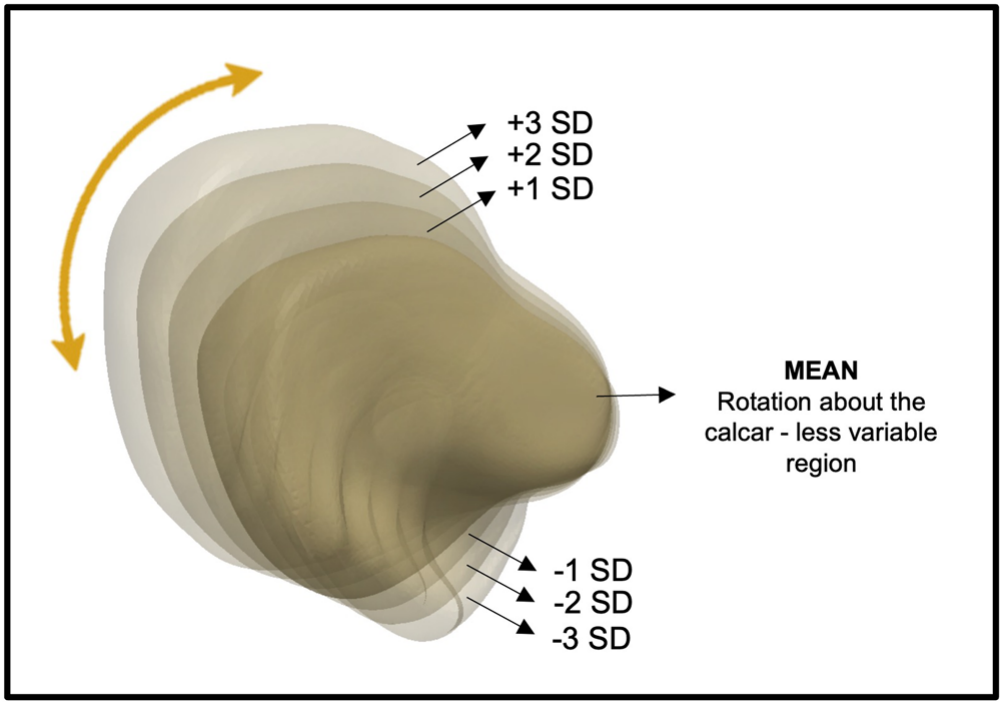
Analysis of principle component 2. An axial view of the three‐dimensional (3D) model of the mean femoral canal shape. As the mean shape was altered by ±3 standard deviation (SD), the femoral canal exhibited proximal femoral torsion around the calcar. Size is also a contributing factor to this principle component, as the proximal region (around the greater trochanter) appears to enlarge in size in conjunction with the rotation.

Rotation about the calcar was identified as the feature corresponding to the variability accounted by PC2 (Figure 6). This showed a torsional change in the proximal end of the canal, which is fixed at the calcar, suggesting that this region is more consistent between the subjects. This mode accounted for 19% of the variance (eigenvalue = 1.9 × 10^5^).

**Figure 7:**
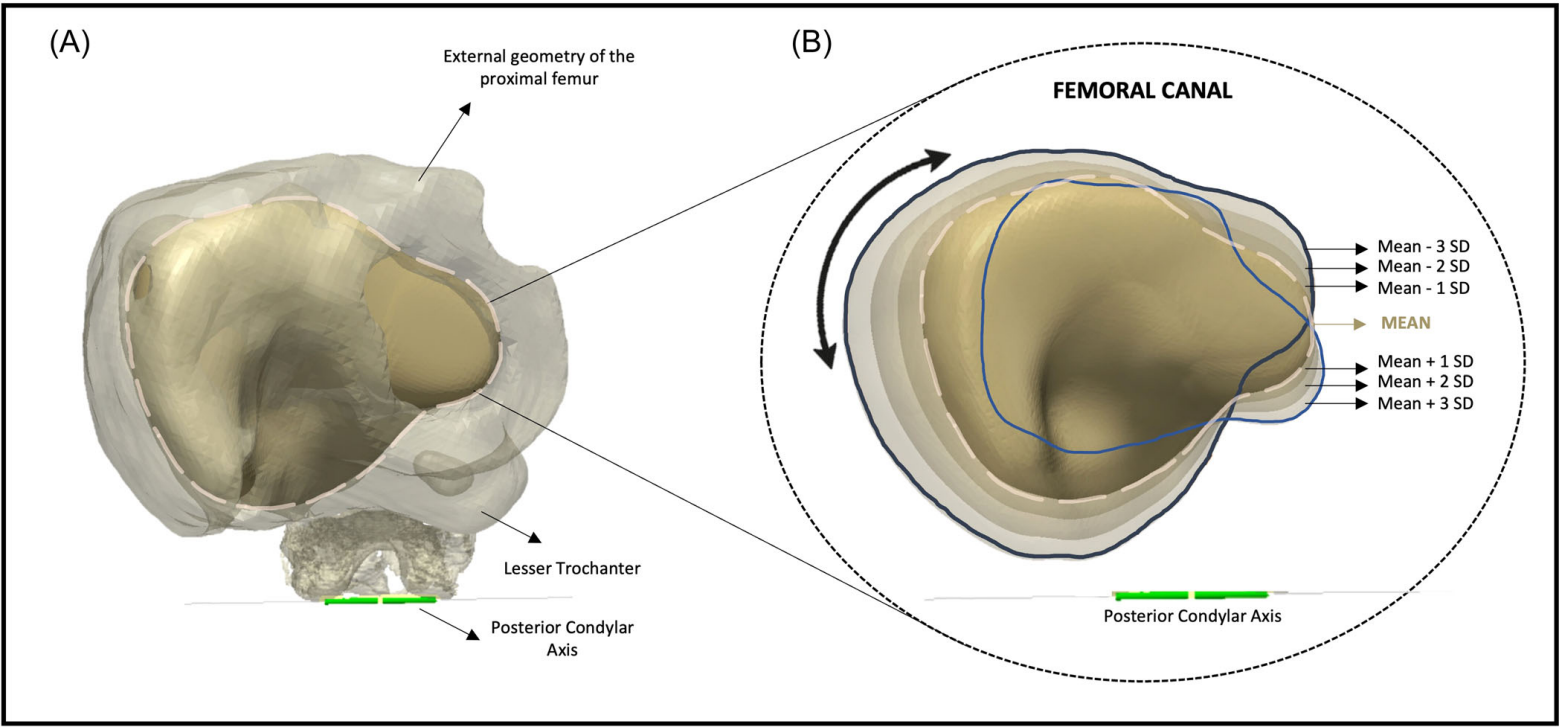
Analysis of principle component 3. (A) An axial view of the 3D mean femoral canal model within the cortex of the proximal femur; (B) Close‐up view of the femoral canal, capturing changes in the shape as the third PC was manipulated by ±3 standard deviation (SD); femoral version was identified as the anatomical characteristic responsible for the majority of the variation in this principle component. The extremities in both directions away from the mean are outlined in black and blue.

The third PC was best studied by tracking the changes that occurred as we applied different weights to this mode, as viewed axially. The seven deformations of the shape at the regular intervals (−3 SD to +3 SD) are presented in Figure 7, which reveals the intramedullary femoral version as the characteristic that is most strongly correlated with this component. This PC accounted for 15% of the variance (eigenvalue = 1.5 × 10^5^).

**Figure 8:**
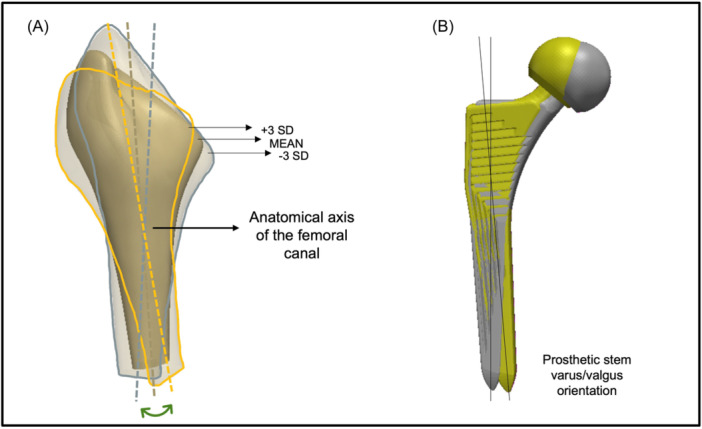
Analysis of principle component 4. (A) As the mean shape was altered by ±3 SD, the varus/valgus orientation of the femoral canal noticeably changed. The orange and blue outlines represent the extremities in both directions away from the mean; (B) The effect of this principle component on the positioning of a femoral stem.

Varus/valgus orientation was found to be the most prominently changing feature associated with the fourth PC and accounted for 9% of the variance (eigenvalue = 8.9 × 10^4^). The seven deformations of the shape as the weights applied to this PC were gradually altered are presented in Figure 8.

**Figure 9:**
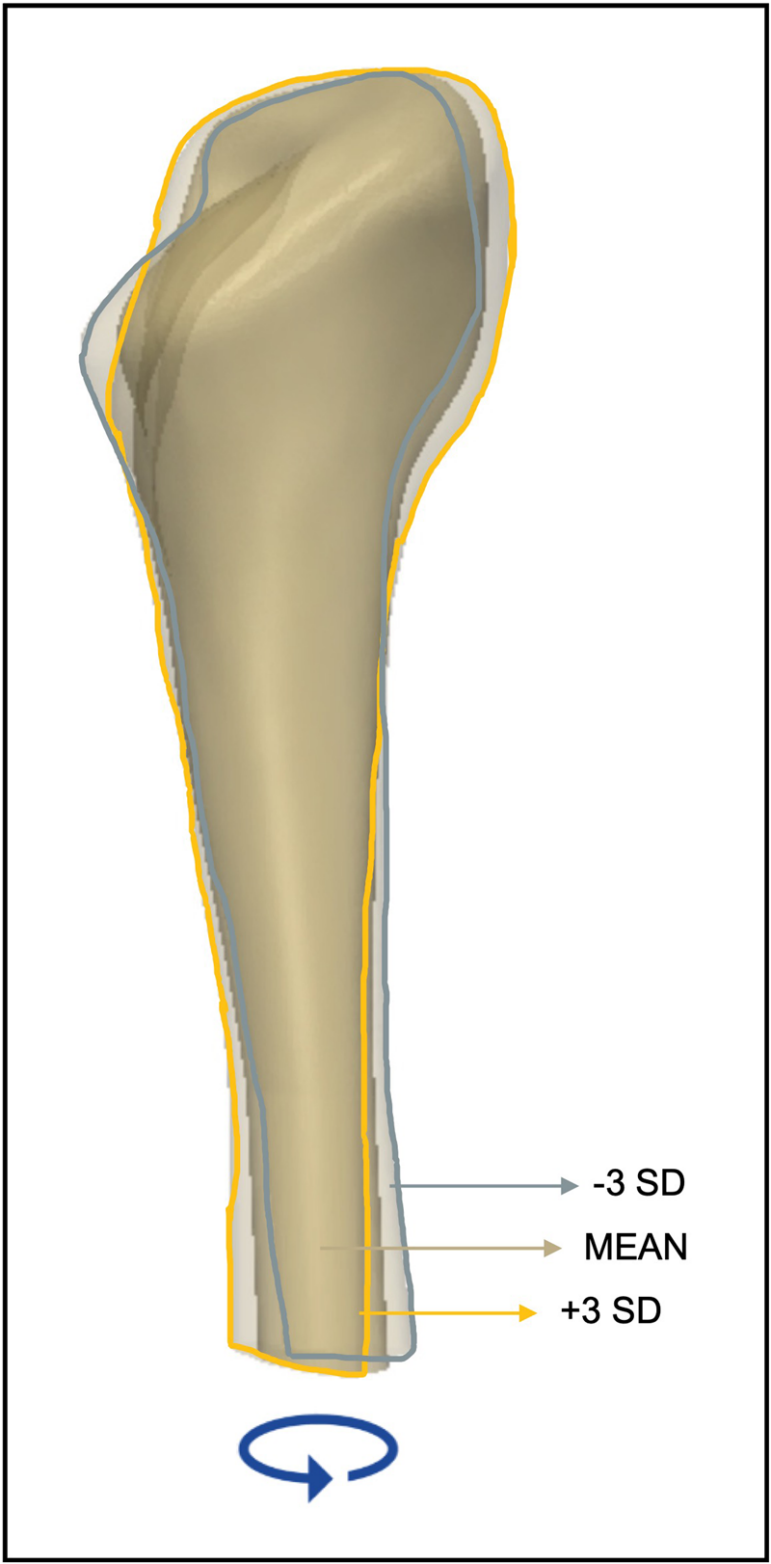
Analysis of principle component 5. As the mean shape was altered by ±3 standard deviation (SD), the distal part of the femoral canal exhibited axial twist/rotation. The orange and blue outlines represent the extremities in both directions away from the mean.

The fifth PC revealed changes mainly in the distal region of the 3D femoral canal models and is summarized as a rotation in the shaft (femoral shaft torsion). This accounted for 6% of the variance (eigenvalue = 6.1 × 10^4^) and governs the distal fixation of the femoral stem (Figure 9).

**Figure 10:**
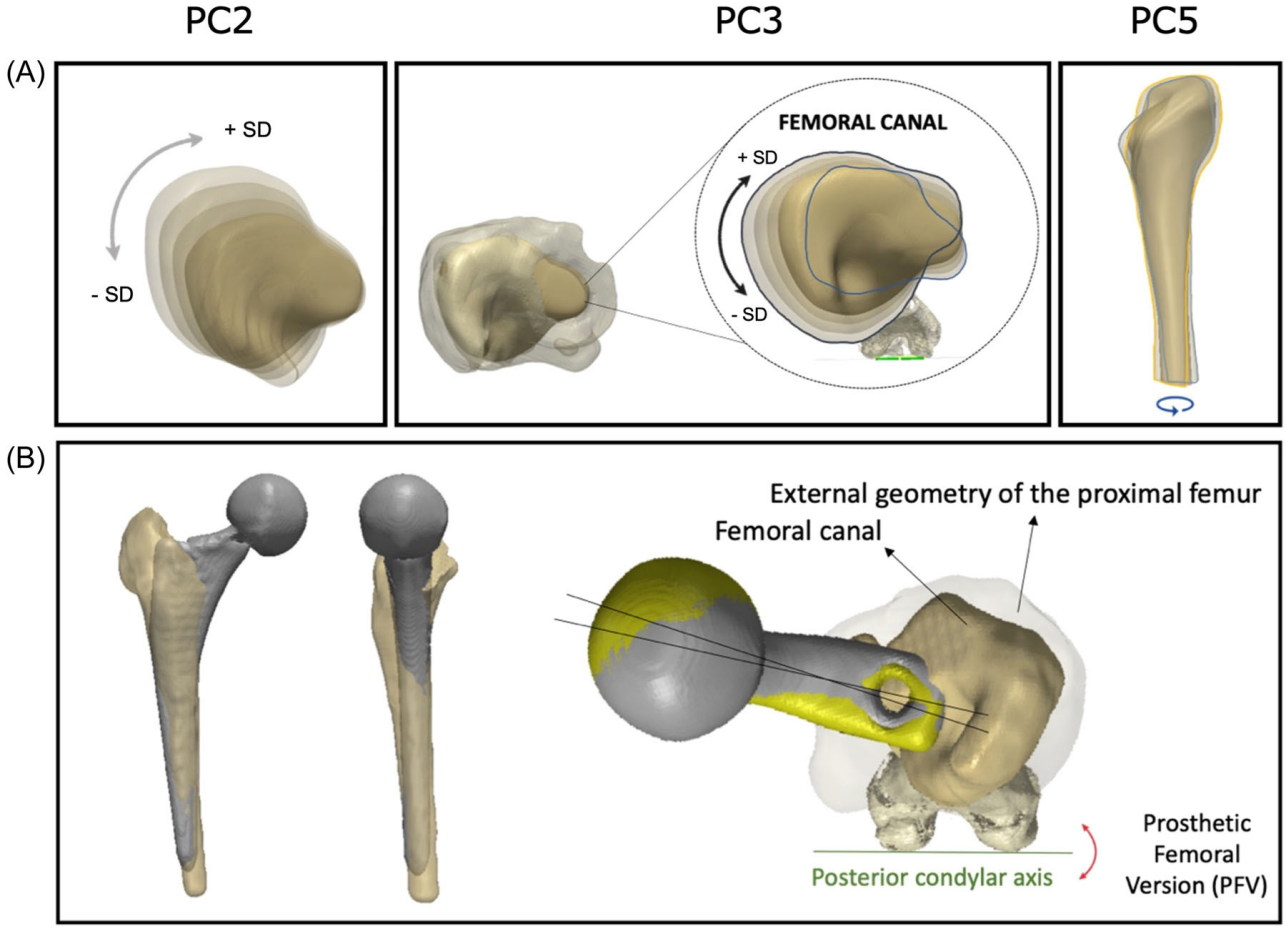
(A) The three identified principle component modes govern the position and orientation of the prosthetic stem and impact the PFV; (B) Anterior‐posterior, sagittal, and axial views of the fit of a prosthetic femoral stem within the medullary canal of the proximal femur.

Visual representations of all five PCs are presented in Figures 4, 5, 6, 7, 8, 9. Based on these findings, the most variable characteristics/features of the intramedullary femoral canal are size (thickness), axial rotation (around the calcar and also around the long axis of the femur), varus/valgus orientation, and femoral shaft torsion. Three of these modes reveal a form of axial twist/rotation, so it is assumed that a combination of these three features affects the PFV of an implanted uncemented stem (Figure 10).

## 4 DISCUSSION

At present, there are no reliable methods to predict the version of femoral stems. The NFV, measured as the angle between the femoral neck axis and the posterior condylar line, has proven to be an unreliable guide. The intraoperative estimation of femoral stem anteversion is typically made by the surgeon through visual assessment of the relative position between the stem and the condylar plane of the femur. This has proven to be an inaccurate approach with poor precision.^9^, ^16^, ^17^ A deviation of up to 44° was measured for the stem version between the intraoperative visual estimation and the postoperative 3D‐CT measurement by Woerner et al.^17^

The NFV for all 64 proximal femurs used in this study was measured. Since this patient cohort has been implanted with an uncemented femoral stem, the achieved stem version was also measured (from the postoperative CT scan) and known, which is guided by the shape of the intramedullary canal. The mean native and stem versions were 16.3 ± 9.4° and 14.7 ± 8.9°, respectively, with a mean difference of 5.5 ± 4.4°. The difference between the measured native and stem version spanned a wide range, from 0.2° to 19.4°. These additional results show that the external geometry does not match the femoral canal shape, indicating that the native femoral version is not a useful guide for predicting the PFV. This further supports the need to model the intramedullary canal during preoperative planning.

This study is the first of its kind to build an SSM on the internal canal of the proximal femur to identify the main modes of variation. Size was identified as the most prevalent mode of the statistical model for all 64 femoral canals. Since only the length of the femoral canals was standardized and not volume/size, this mode predictably captured size as the most prominent variable. However, it was noted that the flare of the femoral canal also played a contributing role to the variability in this first PC. The subsequent modes described angular changes of the proximal and distal end of the canal shape.

The second and third modes of variation exhibited angular changes of the canal in the superior end. Both of these PCs revealed a torsional change, however, the former presents axial rotation, which is fixed at the medial calcar, while the latter is a rotation around the principle axis of the femoral canal (line passing through the center of the femur). The femoral calcar is defined as the densest region of thickened cancellous bone which lies deep to the lesser trochanter and is generally on the inner side of the femur.^18^ Due to the similarity between PC2 and PC3, both visually and also their contribution to the overall variance, they can be considered to have a combined effect on the intramedullary femoral anteversion. While the first PC, size, will help to inform the implant design and component size, these two PCs may help to plan and predict the PFV.

The fourth PC, which was attributed to varus/valgus orientation, can be reasoned by considering the effects of sex on the orientation of the femoral joint line. The work conducted by Springer and colleagues^19^, ^20^ has studied the impact of multi‐factorial characteristics of the patient on the femoral and tibial anatomy. Findings of these studies have established that female patients present with more valgus angulation of the knee joint line when compared with male patients, and a more varus orientation can be found in a male cohort. This trend in the configuration of the knee is also recognized as being a contributing factor to the higher prevalence of valgus osteoarthritis in female patients, along with the overall decreased femoral offset in women.^19^ It can therefore be inferred that since the data set used to train the SSM in this study included femoral canals from both sexes, varus/valgus orientation was identified as one of the principal modes of variation. The shape differences at perturbations of ±3 SD from the mean for this PC may be interpreted as a shift from a more valgus “female” femoral canal to a more varus ‘male’ femoral canal. Other factors are known to influence the varus/valgus orientation of the lower limb, such as ligament imbalances, torsional malalignment of the lower limb, and femoral fractures. None of these were present in the patient cohort included in this study.

The fifth PC of the statistical model described variation at the distal end of the canal. This characteristic of the canal is most commonly understood as the natural anteroposterior and mediolateral bow of the femoral canal.^21^ Due to the limited field of view of the CT scans, the bow and natural twist of the femoral canal are less apparent. This feature is also known to affect the femoral stem anteversion, given that the insertion of a straight and rigid prosthetic stem down a curved and cylindrical structure will cause the stem to jam in a single position. The orthopedic surgeon has very little intraoperative control over this position, which is why the choice of fixation must be determined accurately during the planning process of the surgery.

This study's findings have the potential to enhance the preoperative planning of PFV, making them clinically significant to consider. Making more informed decisions about the fixation type (cemented vs. uncemented) and stem design can be aided by recognizing patterns in the canal shape and the corresponding obtained PFV. When a standard prosthesis is unable to accommodate the anatomic variations in the femoral canal shape, inhibiting primary stability and proximal fixation, custom‐made cementless femoral stems are used. This includes cases where there is a mismatch between the patient's femoral metaphyseal and diaphyseal diameters.^22^ Using the current model to help identify such cases, can make the choice of stem design more efficient.

The shape of the intramedullary canal of the proximal femur is not extensively studied and it does not have the same protruding landmarks and curvature as the external geometry. Knowledge on the shape of the internal canal has previously been retrieved from a plain radiograph, which presents its flaws. Radiographic measurements of indices such as the canal flare index (CFI), taper of the canal, canal–bone ratio (CBR), and canal–calcar ratio (CCR) are prone to errors. This is because any malrotation of the hip will have a direct effect on the measurements, making the results less reproducible when compared with 3D‐CT.^23^, ^24^

Boymans et al.^25^ offer a 3D analysis of the proximal femoral canal morphology using CT scans, to better understand age‐related changes and sex‐related differences in its shape. They acknowledge that a combination of both the cementless stem design and the shape of the canal must be considered when looking at the performance of the prostheses, as a mismatch can lead to inferior performance. The population of interest in this research were older subjects, from a Caucasian origin. Therefore, the findings are not generalizable to other age ranges and ethnicity groups.

In the work conducted by Veilleux et al.^7^ major landmarks of the proximal femur were identified and an SSM was generated from 144 asymptomatic femurs. Using these consistently identified points and major landmarks of the femur, 200 angular/distance parameters were measured and used to predict the femoral version. However, the internal femoral canal was not considered in this study.

This study offers a better understanding of the complex femoral canal shape to the reader. This may, in turn, help to plan the position and orientation of a stem in uncemented THA more accurately, given that the fit of the prosthetic stem is determined by the twist and bow of the canal. The results presented in this study show that there is indeed a variability between the canal shapes of different subjects.

We acknowledge limitations. Due to the size of the data set available, two separate models for both sexes were not generated, and the analysis was based on both altogether (for a larger sample size). Further refinement of the model and access to more CT images can help to better understand the differences between these two subgroups.

Also, analysis of PCs beyond PC5 may be helpful to identify any additional distinguishing features of the femoral canal, which have a lower contribution to the overall cumulative variance. But for the purpose of this study, the major contributing factors to the variation in the population of femoral canals have been identified, which are most likely to impact the achieved PFV and help improve its planning.

The inherently subjective nature of the interpretation of the PCA modes is recognized as another limitation. Due to a lack of consistent protruding landmarks on the femoral canals, feature‐based measurements could not be taken on them. With the use of such quantitative measures, features may be monitored both between and within the various PC modes, allowing for the identification of the shape's most obviously changing characteristic. This would allow us to objectively attribute each PC to a feature as the interdependencies of the PC modes meant that changes in more than one variable were captured by each PC. Further investigations and acquisition of these measurements may be considered in future studies, however, in this study, a team of orthopedic surgeons together with expert engineers came to a mutual consensus regarding the interpretation of the modes.

## 5 CONCLUSION

The intersubject anatomical variability in proximal femoral canal shapes for a population of 64 patients was assessed in the present study, using PCA‐based SSM. This mathematical model provides a valuable tool for analyzing and characterizing geometric shape variations in the femoral canal. The large variations in the size, shape, and orientation of the femoral canals can be holistically captured by a series of PC modes. The 3D models used in this study have shown to be highly variable in terms of these aspects. Since the femoral version was identified as a key changing characteristic, it provides evidence that the variability commonly found in the achieved PFV is largely due to the canal shape into which the stem is fitted.

## AUTHOR CONTRIBUTIONS

Angelika Ramesh contributed to the research design and acquisition, analysis and interpretation of the data. Also drafted and revised the manuscript. Johann Henckel contributed to the research design and acquisition, analysis and interpretation of the data. He has helped to draft and revise the manuscript. Alister Hart contributed to the research design and acquisition, analysis and interpretation of the data, helped to draft and revise the manuscript. Anna Di Laura contributed to the research design and acquisition, analysis and interpretation of the data. She has helped draft and revise the manuscript. All authors have read and approved the final submitted manuscript.

## References

[1] D'Lima DD , Urquhart AG , Buehler KO , Walker RH , Colwell CW . The effect of the orientation of the acetabular and femoral components on the range of motion of the hip at different head‐neck ratios. J Bone Joint Surg‐Am Vol. 2000;82(3):315‐321. 10.2106/00004623-200003000-00003 10724224

[2] Sun J , Zhang Y , Shen J , et al. Comparison of preoperative computed tomography and intraoperative estimation in predicting the version of a single‐wedge femoral stem. Orthop Surg. 2022;14(11):2979‐2986. 36177805 10.1111/os.13524 PMC9627059

[3] Park KK , Tsai TY , Dimitriou D , Kwon YM . Utility of preoperative femoral neck geometry in predicting femoral stem anteversion. J Arthroplasty. 2015;30(6):1079‐1084. 25683295 10.1016/j.arth.2015.01.016

[4] Moralidou M , Di Laura A , Henckel J , Hart AJ . Can version of the proximal femur be used for CT planning uncemented femoral stems? Med Eng Phys. 2023;116:103985. 37230697 10.1016/j.medengphy.2023.103985

[5] Dimitriou D , Tsai TY , Kwon YM . The effect of femoral neck osteotomy on femoral component position of a primary cementless total hip arthroplasty. Int Orthop. 2015;39(12):2315‐2321. 25787684 10.1007/s00264-015-2739-1

[6] Taniguchi N , Jinno T , Koga D , Hagino T , Okawa A , Haro H . Cementless hip stem anteversion in the dysplastic hip: A comparison of tapered wedge vs metaphyseal filling. J Arthroplasty. 2017;32(5):1547‐1552. 28110848 10.1016/j.arth.2016.12.020

[7] Veilleux NJ , Kalore NV , Wegelin JA , Vossen JA , Jiranek WA , Wayne JS . Automated femoral version estimation without the distal femur. J Orthop Res. 2018;36(12):3161‐3168. 30074280 10.1002/jor.24121

[8] Belzunce MA , Henckel J , Di Laura A , Hart A . Uncemented femoral stem orientation and position in total hip arthroplasty: A CT study. J Orthop Res. 2020;38(7):1486‐1496. 32056292 10.1002/jor.24627

[9] Unlu MC , Kesmezacar H , Kantarci F , Unlu B , Botanlioglu H . Intraoperative estimation of femoral anteversion in cementless total hip arthroplasty using the lesser trochanter. Arch Orthop Trauma Surg. 2011;131(9):1317‐1323. 10.1007/s00402-011-1282-9 21359870

[10] Su XY , Zhao Z , Zhao JX , et al. Three‐Dimensional analysis of the curvature of the femoral canal in 426 Chinese femurs. BioMed Res Int. 2015;2015(318391):1‐8. 10.1155/2015/318391 PMC4657382 26640785

[11] Bargar WL , Jamali AA , Nejad AH . Femoral anteversion in THA and its lack of correlation with native acetabular anteversion. Clin Orthop Relat Res. 2010;468(2):527‐532. 19714389 10.1007/s11999-009-1040-2 PMC2806998

[12] Kim KJ , Kim DH , Lee JI , Choi BK , Han IH , Nam KH . Hounsfield units on lumbar computed tomography for predicting regional bone mineral density. Open Med. 2019;14:545‐551. 10.1515/med-2019-0061 PMC6689205 31410366

[13] Singh R , Singh R , Baby B , Suri A . Effect of the segmentation threshold on computed tomography–based reconstruction of skull bones with reference optical three‐dimensional scanning. World Neurosurg. 2022;166:e34‐e43. 35718274 10.1016/j.wneu.2022.06.050

[14] Ramesh A , Di Laura A , De Angelis S , et al. Bone remodeling after revision total hip arthroplasty for large acetabular defects. J Orthop Res. 2024;1‐12. In press 10.1002/jor.25936 38992884

[15] Medacta International . MyHip Preoperative Planning. Medacta International, 1‐19.

[16] Dorr LD , Wan Z , Malik A , Zhu J , Dastane M , Deshmane P . A comparison of surgeon estimation and computed tomographic measurement of femoral component anteversion in cementless total hip arthroplasty. J Bone Joint Surg. 2009;91(11):2598‐2604. 10.2106/JBJS.H.01225 19884433

[17] Woerner M , Sendtner E , Springorum R , et al. Visual intraoperative estimation of cup and stem position is not reliable in minimally invasive hip arthroplasty. Acta Orthop. 2016;87(3):225‐230. 10.3109/17453674.2015.1137182 26848628 PMC4900086

[18] Mei J , Pang L , Jiang Z . Strategies for managing the destruction of calcar femorale. BMC Musculoskelet Disord. 2021;22(460):460. 34011332 10.1186/s12891-021-04324-3 PMC8136139

[19] Springer B , Bechler U , Waldstein W , Rueckl K , Boettner CS , Boettner F . The influence of femoral and tibial bony anatomy on valgus OA of the knee. Knee Surg Sports Traumatol Arthrosc. 2020;28:2998‐3006. 31595340 10.1007/s00167-019-05734-6

[20] Zeng YM , Wang Y , Zhu ZA , Dai KR . Effects of sex and lower extremity alignment on orientation of the knee joint line in knee surgery. Chin Med J. 2012;125(12):2126‐2131. 22884141

[21] Watanabe K , Mitsui K , Usuda Y , Nemoto K . An increase in the risk of excessive femoral anteversion for relatively younger age and types of femoral morphology in total hip arthroplasty with direct anterior approach. J Orthop Surg. 2019;27(2):230949901983681. 10.1177/2309499019836816 30913961

[22] Wera GD , Dwyer MW , Verhotz DR , Popa MA , Marcus RE . Custom cementless femoral stems in total hip arthroplasty. J Hip Surg. 2019;03(2):068‐072.

[23] Casper DS , Kim GK , Parvizi J , Freeman TA . Morphology of the proximal femur differs widely with age and sex: relevance to design and selection of femoral prostheses. J Orthop Res. 2012;30(7):1162‐1166. 22570223 10.1002/jor.22052

[24] Grevenstein D , Vidovic B , Baltin C , et al. The proximal femoral bone geometry in plain radiographs. Archives Bone Joint Surg. 2020;8(6):675‐681. 10.22038/abjs.2020.44937.2226 PMC7718562 33313347

[25] Boymans TAEJ , Heyligers IC , Grimm B . The morphology of the proximal femoral canal continues to change in the very elderly: implications for total hip arthroplasty. J Arthroplasty. 2015;30(12):2328‐2332. 26187385 10.1016/j.arth.2015.06.020

